# Prognostic Value of Machine Learning in Patients with Acute Myocardial Infarction

**DOI:** 10.3390/jcdd9020056

**Published:** 2022-02-11

**Authors:** Changhu Xiao, Yuan Guo, Kaixuan Zhao, Sha Liu, Nongyue He, Yi He, Shuhong Guo, Zhu Chen

**Affiliations:** 1 Hunan Key Laboratory of Biomedical Nanomaterials and Devices, Hunan University of Technology, Zhuzhou 412007, China; hutxch@163.com (C.X.); neseit@163.com (K.Z.); lsdd0789@163.com (S.L.); nyhe@seu.edu.cn (N.H.); 2 Department of Cardiovascular Medicine, Zhuzhou Hospital Affiliated to Xiangya School of Medicine, Central South University, Zhuzhou 412007, China; heyicardiology@163.com (Y.H.); shgzhuzhou@163.com (S.G.); 3 Department of Cardiovascular Medicine, Xiangya Hospital, Central South University, Changsha 410008, China

**Keywords:** acute myocardial infarction, major adverse cardiovascular events, machine learning, logistic regression analysis

## Abstract

(1) Background: Patients with acute myocardial infarction (AMI) still experience many major adverse cardiovascular events (MACEs), including myocardial infarction, heart failure, kidney failure, coronary events, cerebrovascular events, and death. This retrospective study aims to assess the prognostic value of machine learning (ML) for the prediction of MACEs. (2) Methods: Five-hundred patients diagnosed with AMI and who had undergone successful percutaneous coronary intervention were included in the study. Logistic regression (LR) analysis was used to assess the relevance of MACEs and 24 selected clinical variables. Six ML models were developed with five-fold cross-validation in the training dataset and their ability to predict MACEs was compared to LR with the testing dataset. (3) Results: The MACE rate was calculated as 30.6% after a mean follow-up of 1.42 years. Killip classification (Killip IV vs. I class, odds ratio 4.386, 95% confidence interval 1.943–9.904), drug compliance (irregular vs. regular compliance, 3.06, 1.721–5.438), age (per year, 1.025, 1.006–1.044), and creatinine (1 µmol/L, 1.007, 1.002–1.012) and cholesterol levels (1 mmol/L, 0.708, 0.556–0.903) were independent predictors of MACEs. In the training dataset, the best performing model was the random forest (RDF) model with an area under the curve of (0.749, 0.644–0.853) and accuracy of (0.734, 0.647–0.820). In the testing dataset, the RDF showed the most significant survival difference (log-rank *p* = 0.017) in distinguishing patients with and without MACEs. (4) Conclusions: The RDF model has been identified as superior to other models for MACE prediction in this study. ML methods can be promising for improving optimal predictor selection and clinical outcomes in patients with AMI.

## 1. Introduction

Acute myocardial infarction (AMI) is one of the major causes of mortality worldwide [1]. Advancements in healthcare, the survival rate, clinical symptoms, and quality of life after successful percutaneous coronary intervention (PCI) and other therapeutic strategies have improved outcomes for patients with AMI [2]. However, major adverse cardiovascular events (MACEs), including myocardial infarction, heart failure, kidney failure, coronary events, cerebrovascular events, and death, still occur in 20–40% of patients with AMI within 2 years after disease onset [3,4].

Accurate prediction of the incidence of MACEs after AMI, identification of high-risk clinical predictors, and strengthening the management of high-risk patients can improve the long-term prognosis of patients, which may also effectively reduce the rate of MACEs. Traditionally, various scores were used for risk stratification in patients with AMI, such as Thrombolysis in Myocardial Infarction and the Global Registry of Acute Coronary Events [5,6]. However, these traditional methods for evaluating the severity and prognosis of the disease were time consuming, labour-intensive, and inaccurate, which was unsuitable for the assessment of prognosis [7,8,9,10]. Therefore, advanced and precise prediction methods for MACEs in patients with AMI were desperately needed.

Machine learning (ML), as an important branch of artificial intelligence, has a substantial effect on many areas of technology and science [11]. It realizes prediction or decision-making tasks with algorithms and statistical models using computer systems [12]. Depending on the task and type of feedback, ML can be divided into supervised learning, unsupervised learning, and reinforcement learning [13]. Supervised learning is the most widely used ML method in the diagnosis, treatment, and rehabilitation of patients with cardiovascular diseases [14]. In supervised learning, an annotated label is available for each sample and the purpose is to reduce the error between the observed and predictive labels by feeding the model with processed data [15]. In other words, the objective of supervised learning is to determine a function that produces an output based on the corresponding input so as to estimate the annotated label approximately.

The mainstream supervised ML algorithms mainly consist of decision tree (DT), Naive Bayes (NB), support vector machine (SVM), random decision forest (RDF), gradient boosting (GB), multilayer perceptron (MLP) methods, and so on, and different ML models have different characteristics. DT recursively partitions the input space and then fits a local model in each resulting region of the input space, but it tends to deliver high variance estimators in that they are unstable [16]. NB is a probabilistic classifier that assumes the input variables are conditionally independent given the class label and requires few data to estimate the small number of parameters, making NB relatively immune to overfitting [17]. SVM attempts to find an optimal hyperplane in an N-dimensional input space that maximizes the distance between the data points of the two classes and is famous for its generalization performance and ability to handle high dimensional data [18]. RDF is an ensemble learning method that built on DT. In order to address the drawback of DT, it uses a technique referred to as bagging to reduce the variance of an estimate by averaging together many estimates. RDF has high prediction accuracy, good tolerance to outliers and noise, and is not prone to overfitting [19]. GB is another ensemble learning algorithm also based on DTs. Unlike RDF, a method known as boosting converts weak learners into strong learners while reducing the bias of the model at each round, which results in accurate predictions [20]. MLP is a class of models that can serve as universal function approximators capable of modelling non-linear interactions between features. They can meet the requirements of solving accuracy and generalization with lower informational inputs [21]. The research on ML algorithms has developed rapidly and is widely used in practice.

Recently, ML has been widely used in various aspects of the management of cardiovascular diseases [11], such as rapid diagnostics, precise treatment, and prognostication, including the prediction of mortality and other adverse prognoses. Liu et al. [22] found that ML models showed marginal value in improving the prediction of 30-day MACEs for emergency department chest pain patients and finally developed the best multidimensional scaling algorithm, with an area under the curve (AUC) of 0.901. Khera et al. [23] drew the conclusion that ML offered resolution for high-risk AMI individuals and reported the best meta-classifier with an AUC of 0.90. Overall, the use of ML has resulted in improved management of cardiovascular diseases [24,25]. ML also has great potential for predicting MACEs in patients with AMI [22,26]. However, the comparison or combination of different algorithms may increase the predictive accuracy for diseases, which deserves further investigation so as to improve the prognoses of patients with AMI.

Thus, in this study, we explored the independent predictors of MACEs in patients with AMI by LR analysis using medical records from the hospital information system. Moreover, the performance of traditional LR was compared with those of the optimized DT, NB, SVM, RDF, GB, and MLP approaches. The aim of this study was to explore predictors of MACEs in patients with AMI and identify the most appropriate algorithm for prediction by comparing six ML algorithms with traditional LR analysis.

## 2. Materials and Methods

### 2.1. Study Population and Statistical Analysis

**Figure 1:**
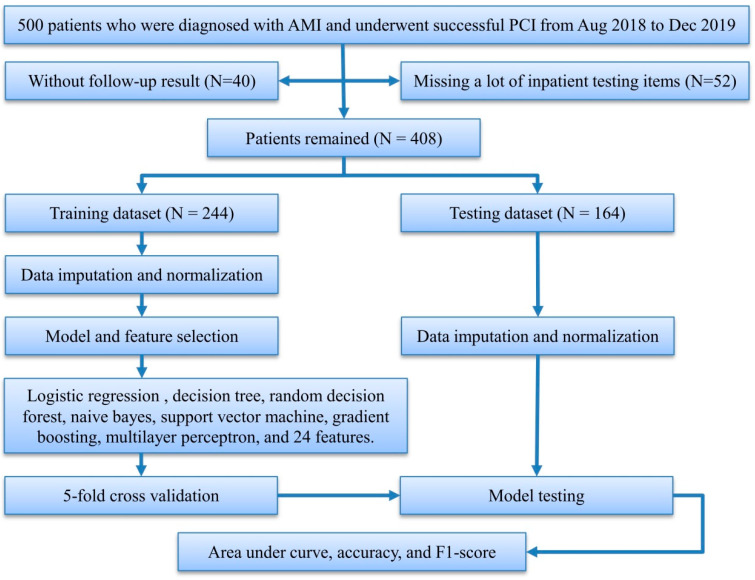
Study flowchart. AMI, acute myocardial infarction; PCI, percutaneous coronary intervention.

We initially collected 500 patients who were enrolled in the hospital information system of Zhuzhou Central Hospital from August 2018 to December 2019, underwent coronary angiography and successful PCI, and were diagnosed with AMI, after excluding patients who did not receive standard treatment and died during hospitalization, and followed up these patients during November to December 2020. Forty patients who were lost to the follow-up and 52 patients without necessary in-patient testing items were excluded. In all, 408 patients were included for the prediction of MACEs and the mean follow-up time was calculated as 1.42 years (Figure 1). 

Subsequently, the collected raw data was translated into our structured database for further analysis. The continuous variables were recorded as means ± standard deviation, and categorical variables were recorded as the sample rate. SPSS Statistics version 20 (IBM Co. Ltd., Armonk, NY, USA) was used for statistical analysis. Statistical differences of continuous variables were calculated using the Student’s *t*-test, and differences of categorical variables were evaluated using a chi-squared test. Significance was set at *p* < 0.05.

### 2.2. Preprocessing and Feature Selection

The structured data was divided into training and testing datasets in a ratio of six to four, and imputation and normalization of data were carried out for these datasets, respectively. Since data distribution characteristics differed between categorical and continuous variables, different methods were used to impute missing values. For a categorical variable, the proportion of each category in the existing values was calculated, and missing values were imputed with a set of categories which retained the same categorical proportion as the existing values. A continuous variable was imputed with random numbers that were generated by Gaussian distribution, the mean and standard deviation of which were calculated from the existing values. If the random numbers were out of the range of the existing values, the missing values were imputed with the mean of the existing values. Due to the wide distribution range of the diverse predictors and some of the prediction models used requiring the normalization of values, the min–max scaler was used after data imputation.

The structured dataset consisted of 41 clinical variables (Table S1), and feature selection was performed only on the training dataset. First, features with missing values greater than 20% were discarded, and discrete variables whose variance did not meet the set threshold of 0.09 were removed according to the calculation method of variance for Bernoulli random variables. Second, recursive feature elimination with random decision forest (RFE-RDF) identified the optimal number of features.

### 2.3. LR Analysis

This study was designed to judge whether the AMI patient has MACEs or not, which is discrete. To further assess the association between occurrence of MACEs and the selected features, an LR analysis [27] performed with discrete cutoffs was used to determine the independent predictors of MACEs, then odds ratio (OR) and significant differences were recorded to evaluate the prognostic relevance of these features.

### 2.4. Model Development

By using patients’ medical records, those with AMI were randomly divided into two groups, as mentioned earlier: 60% of the patients were assigned to model development and 40% to testing. Hyperparameters were optimized using five-fold cross-validation in five ML (including DT, NB, SVM, RDF, and GB) and LR models, and a manual grid search was used for parameter optimization in the rest of the MLP models (Table S2). The training dataset was divided into five exclusive subsets by the five-fold cross-validation, using four for model development and the remainder for model validation. This process was repeated five times. The validation datasets were used for assessing the performance (such as AUC, accuracy, and F1-score) of all ML models. In the manual grid search, some parameters of models, such as epoch, batch size, learning rate, momentum, activation function, rate of dropout, and the number of hidden layer neurons, were tuned.

Unbalanced data is a common issue in dichotomous classification which results in the models having poor sensitivity. To address class imbalances, we provided a different weight that was inversely proportional to class frequencies in the training dataset for each class. All analyses were performed using Anaconda3-5.1.0-Windows. LR, DT, RDF, NB, SVM, and GB were implemented using scikit-learn v0.19.1. MLP was implemented using Keras v2.2.4. 

### 2.5. Model Testing

To evaluate the models’ performance for the prediction of MACEs, the accuracy, F1-score, and AUC were calculated in the testing dataset. The probabilities of these models were calibrated using Platt’s scaling, and the calibration was measured by the Brier score.

To further identify the discriminative ability among all ML and LR models, the patients were initially divided into two subgroups according to predictive probability. The first subgroup of patients were predicted with good prognosis (predictive probability was less than 0.5) and the second was predicted with poor prognosis (predictive probability was greater than 0.5). The cumulative survival of MACEs was compared across the above groups using Kaplan–Meier analysis. In addition, the patients in the testing dataset were randomly divided into equal subgroups to verify existing survival differences. Finally, all the patients in the testing dataset were analyzed by Kaplan–Meier to assess the incidence of MACEs.

## 3. Results

### 3.1. Clinical Characteristics of the Selected Patients

A total of 408 patients were included in our database and 258 (63.2%) of them were diagnosed as ST-segment elevated myocardial infarction. In total, MACEs occurred in 125 (30.6%) patients after a mean of 1.42 years follow-up. A comparison of the MACE and non-MACE groups is shown in Table 1. On the one hand, the patients in the MACE group were older, had more coronary lesion vessels and greater left ventricular diameters, and showed higher Killip classification and levels of creatinine and uric acid compared with the non-MACE group. On the other hand, the patients in the MACE group exhibited lower levels of cholesterol, low-density lipoprotein, and left ventricular ejection fractions. In addition, MACEs were closely related to past medical history and drug compliance, and patients in the MACE group suffered from other diseases and took medicine irregularly.

### 3.2. The Selected and Independent Predictors

**Figure 2:**
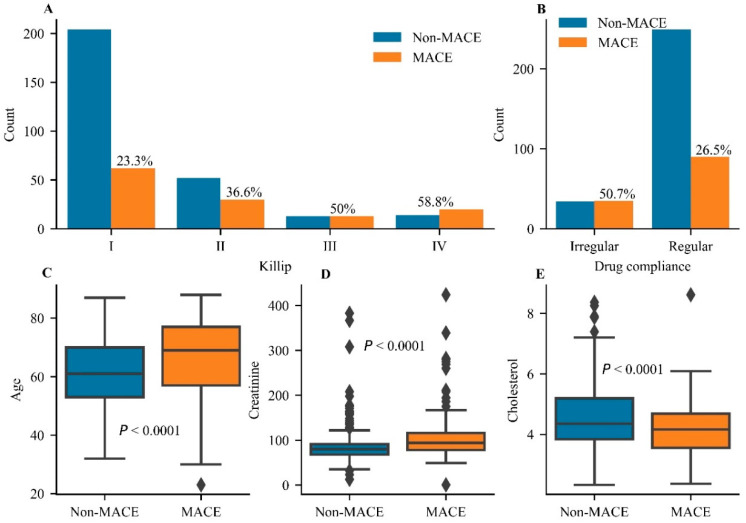
The statistical analysis for independent predictors of major adverse cardiovascular events. (**A**) The comparison of major adverse cardiovascular event (MACE) rates in different grades of the Killip classification. (**B**) The comparison of MACE rates between irregular and regular groups of drug compliance. Analysis of differences for the distribution of age (**C**) and the levels of creatinine (**D**) and cholesterol (**E**) between the Non-MACE group and the MACE group. A significant difference was set at *p* < 0.05.

Twenty-four predictors were retained and no feature was excluded after RFE-RDF (Figure S1 in Supplementary Material). As shown in Table 2 and Figure 2, Killip classification (Killip IV class vs. I class, odds ratio 4.386, 95% confidence interval 1.943–9.904), drug compliance (irregular vs. regular compliance, 3.06, 1.721–5.438), age (per year, 1.025, 1.006–1.044), creatinine (1 µmol/L, 1.007, 1.002–1.012), and cholesterol (1 mmol/L, 0.708, 0.556–0.903) were independent predictors of MACEs. The MACE rate gradually increased from Killip I to IV, suggesting that it was positively related to cardiac function (Figure 2A). Taking drugs regularly reduced the MACE rate to 26.5% in comparison with the rate in those who took drugs irregularly (Figure 2B). Age and the level of creatinine exhibited significant differences in distribution between the MACE and non-MACE groups (Figure 2C,D), suggesting a positive correlation of these variables with the MACE rate. The level of cholesterol showed a negative correlation with the MACE rate (Figure 2E). Overall, Killip classification, drug compliance, age, and levels of creatinine and cholesterol were considered as independent predictors of MACEs and showed better predictive value than others. 

### 3.3. Comparative Performance between the ML and LR Models

**Figure 3:**
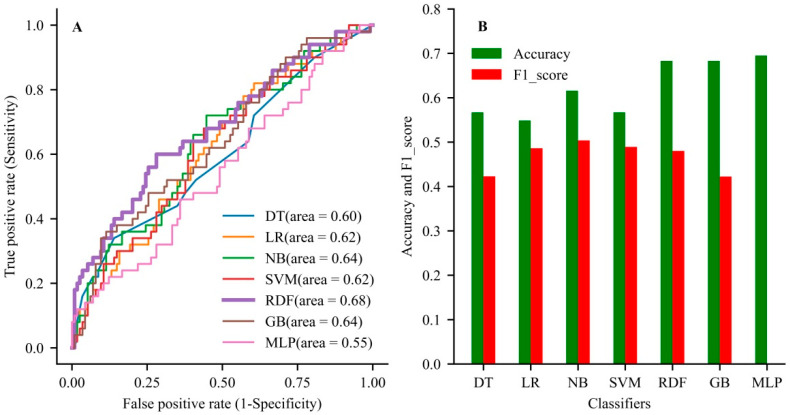
The comparison of generalization ability among the developed models. (**A**) The area under the curve for all models used in the testing dataset. (**B**) The evaluation of accuracy rate and F1-score among the used models in the testing dataset. DT, decision tree; LR, logistic regression; NB, Naive Bayes; SVM, support vector machine; GB, gradient boosting; MLP, multilayer perceptron.

In the validation dataset, the RDF had the best discrimination with an AUC of 0.749 (95% CI, 0.644–0.85), accuracy of 0.734 (0.647–0.820), and F1-score of 0.480 (0.358–0.603) among all models (Table 3). In the testing dataset, the RDF also showed the better generalization ability with an AUC of 0.68, accuracy of 0.68, and F1-score of 0.48 compared with the other models (Figure 3 and Table S3 in Supplementary Material). The RDF showed better performance than the other models when comprehensively evaluating the accuracy and AUC value of the model.

### 3.4. Calibration Plots for All Models

**Figure 4:**
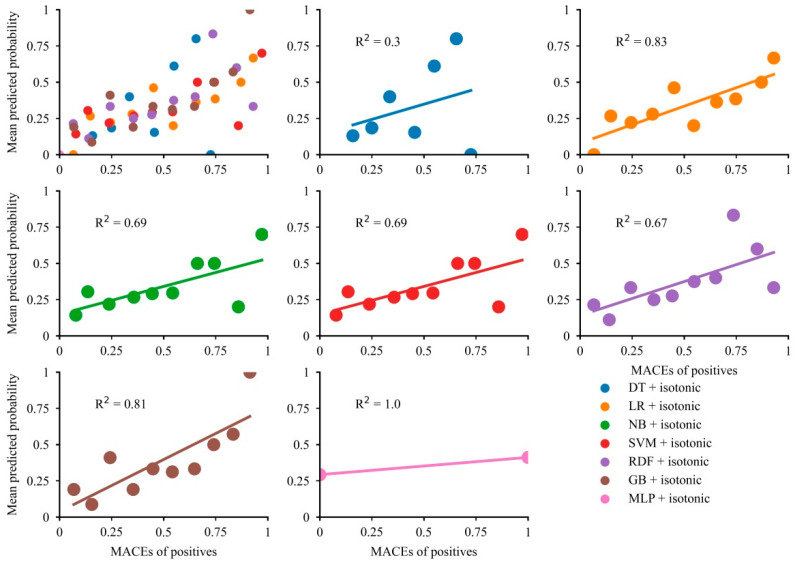
Calibration plots for the developed models.

Overall, the majority of models showed modest concordance between the mean predictive probability and fraction of MACEs (Figure 4). The calibration curves indicated good alignment between the mean predictive probability and fraction of MACEs in LR and GB (*R*^2^ = 0.83 for LR and *R*^2^ = 0.81 for GB), while the predictive probability of MACEs in LR was limited below 0.75. The NB, SVM, and RDF models showed modest calibration. Although the predictive probabilities for the DT model showed a wide range, the calibration curve showed poor concordance. The predicted probability of MLP was a range of 0.25 to 0.45, so the effect of model calibration is very poor. After calibration, the Brier score became smaller, suggesting that the models were well calibrated (Table S4). 

### 3.5. Kaplan–Meier Analysis for MACEs

**Figure 5:**
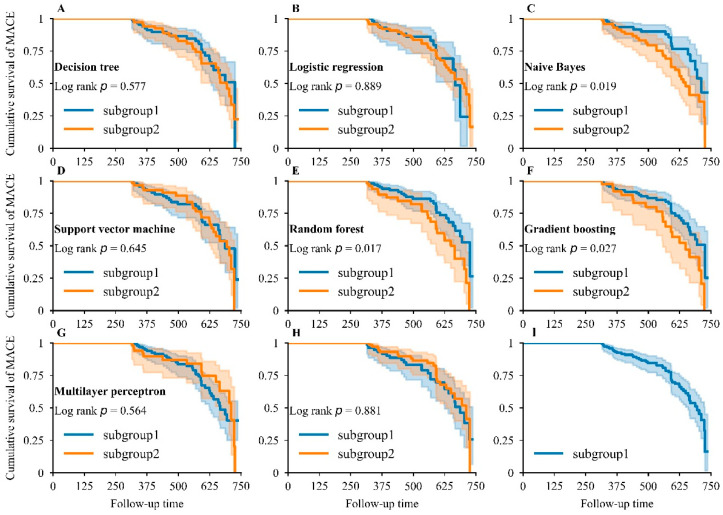
Kaplan–Meier analysis for major adverse cardiovascular events. Kaplan–Meier curve for MACEs in DT (**A**), LR (**B**), NB (**C**), SVM (**D**), RDF (**E**), GB (**F**), and MLP (**G**) models: subgroup1—patients who were predicted to show a good prognosis by the corresponding model; subgroup2—patients who were predicted to show a bad prognosis by the corresponding model. Kaplan–Meier curve for MACEs when the study population in the testing dataset was randomly partitioned into two equal groups (**H**) and when the whole study population in the testing dataset was included (**I**).

The NB, RDF, and GB showed significant differences in survival distribution (Figure 5C,E,F) across two subgroups, and RDF showed the smallest *p*-value (log-rank *p* = 0.017) and was the most appropriate model for distinguishing MACEs. However, LR, DT, SVM, and MLP did not exhibit obvious differences in survival distribution (Figure 5A,B,D,G). In random subgroups, no striking difference was found in survival distribution (Figure 5H), indicating that our developed models had strong discrimination ability. The cumulative survival of patients dropped sharply, highlighting the poor prognosis in patients during an average follow-up period of 1.42 years (Figure 5I).

## 4. Discussion

In this study, we identified five independent predictors that were closely related to adverse prognoses of AMI, namely, Killip classification, drug compliance, age, and levels of creatinine and cholesterol. Among these, Killip classification was considered as the most important predictor since the predictive accuracy increased obviously with the inclusion of this feature in all models. In addition, we developed and validated six ML models to predict MACEs in patients with AMI after the onset of an average follow-up period of 1.42 years, and compared these six ML models with LR to determine the predictive effect of these ML models. Among the ML models, the RDF model showed greater predictive and discriminative advantages compared to the other models.

Identification of independent predictors of MACEs guides and assists precise treatment and rehabilitation and improves the prognoses of patients. In this study, Killip classification, drug compliance, age, and levels of creatinine and cholesterol were identified as independent predictors of MACEs. These predictors could help accurately predict the prognoses of AMI patients and thereby strengthen individualized treatment for patients, such as taking medicine regularly and regular subsequent visits. Li et al. [10] recruited 87 patients with AMI to explore the value of S100A1 for early diagnosis and prognostic assessment. The results showed that a higher concentration of plasma S100A1 was notably associated with a poor prognosis for patients after the first PCI. Topal et al. [28] used 1603 patients to estimate the influence of age on long-term prognosis in ST-segment elevation myocardial infarction and concluded that the risk of death and re-hospitalization depended on both advanced age and infarct size. These results are partly consistent with our findings, although we identified five independent predictors for MACE prediction in this study, which could more effectively avoid the deviations caused by insufficient indicators. Many of these predictors of AMI outcomes have been discussed in previous studies, including creatinine, age, and Killip classification [29,30,31,32]. 

Despite the importance of MACE prediction, its application is still limited in clinical practice [33], necessitating more advanced methods to improve the prediction of prognoses in patients with AMI. Sax et al. [34] used ML to develop a risk-stratification tool for emergency department patients with acute heart failure and they concluded that the use of an XGBoost classifier (AUC of 0.85) improved 30-day risk prediction in comparison with LR. However, their primary outcomes were 30-day serious adverse events, and the applicability of the tool for longer follow-up periods was unknown. Thus, using the medical records of patients with AMI after the onset of an average follow-up period of 1.42 years, we have established six ML models and compared them with LR to identify an appropriate model for MACE prediction. The ML models showed advantages of high precision, automation, rapid response, and the ability to process large-scale data simultaneously [35,36], and could integrate electronic medical data and provide quick predictive outcomes and personalized rehabilitation programs based on the prognostic predictors. The application of ML methods in clinical practice has been also found to improve the predictive accuracy of MACEs when compared with the traditional LR model, which can be used to assist diagnosis and treatment of cardiovascular disease and improve the outcomes of patients.

We found that the RDF produced the better generalization ability compared with the rest of the ML models even though the sample size in our dataset was small. RDF is an ensemble learning algorithm that comprehensively evaluates the results of multiple DTs. Parts of random predictors and samples are selected to train each single tree, after which a final response measurement is generated by vote. When predicting the prognosis of a patient, each DT in the RDF will make a decision to obtain the classification. Through the statistics of the decision results, the classification with the highest number of votes will be determined as the result of the prognosis. RDF can be trained in parallel and has strong generalization ability for prediction of cardiovascular disease. Zhang et al. [37] used the clinical data of AMI patients to predict 30 days of all-cause hospital readmissions, and they found that the AUC value of the RDF model for discriminating cases from controls was 0.701. Yeung et al. [38] developed an RDF model to predict the death of patients with left ventricular thrombus and the results showed that the RDF model achieved an AUC of 0.700 (95% CI 0.553–0.863) on a validation dataset. These studies showed that RDF could be a good model for cardiovascular disease evaluation, which is consistent with our findings. Distinctively, the RDF model in our study showed greater discrimination and its performance has also been compared with other ML and the LR models, which may yield more authentic and reliable results. 

AUC is an essential indicator to evaluate the performance of ML models, which could be affected by various factors. Generally, the original dataset is a decisive factor for the value of AUC, and a dataset of high quality and a larger sample size guarantees an authoritative AUC, otherwise missing values or unbalanced data may lead to a lower AUC [39,40]. Additionally, factors resulting in false negative errors also produce a lower AUC value, especially in predictive studies; for example, a shorter follow-up time means a lower probability for a model to learn positive events and thereby causes data unbalance and false negative errors [41]. Moreover, overfitting for ML models is still a common problem, which may result in failure to generate true predictions for unseen datasets and lead to lower AUC values [42]. In this study, the AUC of the studied ML models is modest, which may be caused by missing values, outliers, sample size, and follow-up time. Therefore, we have used AUC and accuracy together with F1-score to evaluate the reliability of models, which could be more objective in predicting MACEs in patients with AMI.

## 5. Limitations

This study had some limitations. The sample size for model development was not large enough, which may have affected the AUC and accuracy and discrimination of prediction, requiring validation with a larger dataset in future studies. Moreover, the dataset in this study came from a single medical center and the representativeness of the research data may also be limited, calling for further investigation.

## 6. Conclusions

AMI, as the most severe manifestation of coronary artery disease, is a threat to human life all over the world. Precise and quick assessment of MACEs in patients with AMI could improve the prognoses of patients. Therefore, based on the medical records from the hospital information system, we demonstrated that Killip classification, drug compliance, age, and levels of creatinine and cholesterol were independent predictors of MACEs. Although the performance of RDF for MACE prediction was not great after comprehensive evaluation, it was superior to LR and other models, which could provide sufficient predictive performance for MACEs in patients after AMI in this study. Overall, the ML methods are promising tools for cardiovascular disease management and the prognostication of patients by increasing predictive accuracy.

## Figures and Tables

**Table 1:** Patients’ characteristics of included subjects.

Characteristic	All (*N* = 408)	Non-MACEs (*N* = 283)	MACEs (*N* = 125)	*p*-Value
Age (y)	62.95 ± 12.98	61.32 ± 12.32	66.65 ± 13.71	<0.0001
Male (%)	317 (77.7%)	223 (78.8%)	94 (75.2%)	0.421
Follow-up time (d)	516.45 ± 137.82	509.88 ± 139.37	531.30 ± 133.61	0.148
Coronary lesion vessels (num)	2.14 ± 1.00	2.08 ± 1.00	2.27 ± 0.98	0.081
Number of stents implanted	1.79 ± 1.06	1.74 ± 1.08	1.90 ± 1.00	0.145
Electrocardiogram (%) (ST segment elevation)	258 (63.2%)	179 (63.3%)	79 (63.2%)	0.992
Killip classification (I:II:III:IV)				<0.0001
I	266 (65.2%)	204 (72.1%)	62 (49.6%)	
II	82 (20.1%)	52 (18.4%)	30 (24.0%)	
III	26 (6.4%)	13 (4.6%)	13 (10.4%)	
IV	34 (8.3%)	14 (4.9%)	20 (16%)	
Cholesterol (mmol/L)	4.44 ± 1.04	4.56 ± 1.07	4.16 ± 0.93	<0.0001
Low density lipoprotein (mmol/L)	2.68 ± 0.87	2.76 ± 0.87	2.49 ± 0.83	0.004
C-reactive protein (mg/L)	18.14 ± 19.97	17.02 ± 19.49	20.70 ± 20.89	0.086
Left ventricular diameter (mm)	47.18 ± 5.85	46.80 ± 5.39	48.04 ± 6.73	0.049
Left ventricular ejection fraction (%)	0.52 ± 0.10	0.53 ± 0.09	0.50 ± 0.11	0.002
Creatinine (µmol/L)	92.59 ± 46.70	85.63 ± 38.40	108.35 ± 58.68	<0.0001
Uric acid (µmol/L)	349.94 ± 101.27	337.86 ± 98.94	377.29 ± 101.54	2.64 × 10^−4^
Glucose (mmol/L)	8.78 ± 5.14	8.53 ± 5.13	9.36 ± 5.13	0.131
White blood cells (10^9^/L)	10.46 ± 4.29	10.21 ± 4.00	11.02 ± 4.85	0.103
Neutrophils (10^9^/L)	8.07 ± 4.11	7.89 ± 4.03	8.49 ± 4.28	0.168
Hypertension (%)	228 (55.9%)	153 (54.1%)	75 (60%)	0.206
Cigarettes (%)	199 (48.8%)	145 (51.2%)	54 (43.2%)	0.134
Past medical history (%)	132 (32.4%)	83 (29.3%)	49 (39.2%)	0.049
Diabetes (%)	101 (24.8%)	66 (23.3%)	35 (28%)	0.313
Drug compliance (%)	339 (83.4%)	249 (88.0%)	90 (72%)	<0.0001
Revascularization time (min)	4885 ± 11,126	4736 ± 10,264	5224 ± 12,917	0.683
Number of diseased vessels	2.14 ± 1.00	2.09 ± 1.01	2.26 ± 0.98	0.100

**Table 2:** The result of logistic regression analysis.

Characteristic	OR	95% Confidence Interval	*p*-Value
Killip classification III vs. I IV vs. I			0.001
	2.849	(1.181–6.873)	0.02
	4.386	(1.943–9.904)	<0.0001
Drug compliance (irregular vs. regular)	3.06	(1.721–5.438)	<0.0001
Age (per year)	1.025	(1.006–1.044)	0.01
Creatinine (1 µmol/L)	1.007	(1.002, 1.012)	0.004
Cholesterol (1 mmol/L)	0.708	(0.556–0.903)	0.005

**Table 3:** Comparison of performance between various models in the validation dataset.

Classifier	AUC, Mean (95%CI)	Accuracy, Mean (95%CI)	F1-Score, Mean (95%CI)
Logistic regression	0.717 (0.692–0.743)	0.721 (0.648–0.795)	0.565 (0.483–0.646)
Decision tree	0.664 (0.488–0.840)	0.644 (0.463–0.825)	0.532 (0.374–0.690)
Naive Bayes	0.733 (0.650–0.718)	0.742 (0.681–0.803)	0.503 (0.358–0.648)
Support vector machine	0.717 (0.687–0.746)	0.725 (0.639–0.811)	0.570 (0.483–0.656)
Random forest	0. 749 (0.644–0.853)	0.734 (0.647–0.820)	0.480 (0.358–0.602)
Gradient boosting	0.737 (0.637–0.838)	0.729 (0.628–0.831)	0.453 (0.206–0.701)
Multilayer perceptron	0.663 (0.532–0.794)	0.697 (0.599–0.795)	0.103 (−0.162–0.368)

## Data Availability

Data cannot be made available.

## Supplementary Materials

The following are available online at https://www.mdpi.com/article/10.3390/jcdd9020056/s1, Figure S1: Clinical feature selection, Table S1: Detailed information about the collected clinical features, Table S2: Hyper-parameter optimization using five-fold cross-validation, Table S3: Predictive performance of developed machine learning models and logistic regression in the test dataset, Table S4: The Brier score for model calibration.
