# Supplementary material for: Prognostic Value of Machine Learning in Patients with Acute Myocardial Infarction
Source: J Cardiovasc Dev Dis. 2022 Feb 11;9(2):56. doi: 10.3390/jcdd9020056 (PMC8880640; doi:10.3390/jcdd9020056)
Supplement: Supplementary file 1 [file jcdd-09-00056-s001.zip › jcdd-1507648-supplementary.pdf]

Supplementary Material:

Prognostic value of machine learning in patients with acute myocardial infarction

Changhu Xiao[1], Yuan Guo[1, 2, 3]*, Kaixuan Zhao[1], Sha Liu[1], Nongyue He[1], Yi He[2], Shuhong Guo[2], Zhu Chen[1]*

[1]Hunan Key Laboratory of Biomedical Nanomaterials and Devices, Hunan University of Technology, Zhuzhou 412007, Hunan, PR China.

[2]Department of Cardiovascular Medicine, The Affiliated Zhuzhou Hospital Xiangya Medical College, Central South University, Zhuzhou 412000, Hunan, PR China.

[3]Department of Cardiovascular Medicine, Xiangya Hospital, Central South University, Changsha 410008, Hunan, China.

***Corresponding authors:**

Y. Guo, E-mail: guoyuan8141@csu.edu.cn

Z. Chen, E-mail: chenzhu220@163.com

1. Figures

Figure S1. Clinical feature selection

Recursive feature elimination with random decision forest was used to determine whether any subset of inputted features could be excluded. Once any one feature is removed, the area under the carve (AUC) is changed significantly in random decision forest, and the model discrimination is reduced in the test dataset.

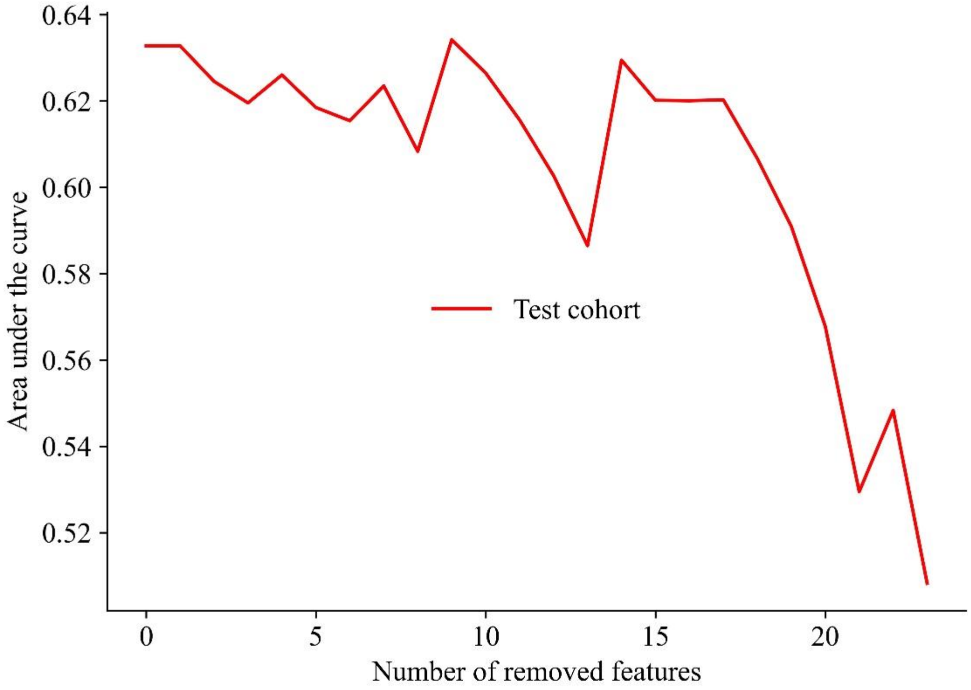

2. Tables

Table S1. Detailed information about the collected clinical features

Features	Status	Features	Status	Features	Status
Age, y	√	Glucose, mmol/L	√	Troponin I, ng/L	×
Gender	√	White blood cells, 10^9/L	√	Creatine kinase isoenzyme, μg/L	×
Follow-up time, d	√	Neutrophils, 10^9/L	√	Creatine kinase, μg/L	×
Coronary lesion vessels, num	√	Hypertension	√	ApoA1, g/L	×
Number of stents implanted	√	Cigarettes	√	D-dimer, ng/mL	×
Electrocardiogram, (ST segment elevation)	√	Past medical history	√	Recurrence of myocardial infarction	×
Killip classification, %	√	Diabetes	√	Regularly subsequent visit	×
Cholesterol, mmol/L	√	Drug compliance	√	Early onset family history	×
Low density lipoprotein, mmol/L	√	Revascularization time, min	√	Dyslipidemia	×
C-reactive protein,	√	Number of	√	Cerebral apoplexy	×

mg/L		diseased vessels			
Left ventricular diameter, mm	√	Onset time of chest pain, h	×	Size of left atrium, mm	×
Left ventricular ejection fraction, %	√	Other diseased vessels, num	×	Saccharification, %	×
Creatinine, μmol/L	√	B-type natriuretic peptide, pg/mL	×	Triglyceride, mmol/L	×
Uric acid, μmol/L	√	Troponin T, μg/L	×		

√: The retained features, ×: The removed features.

Table S2. Hyper-parameters optimization using 5-fold cross validation

	Hyper-parameter space	Best combination of Hyperparameters	AUC in validation set: mean (95% CI)
Random decision Forest	{'criterion': ['gini','entropy'], 'n_estimators': range (1,100), 'max_depth': range (1,100), 'min_samples_split':range(2,10), 'min_samples_leaf':range(1,10), 'max_features': [range(2,25)]}	{'criterion': 'gini', 'n_estimators':50, 'max_depth': 12, 'min_samples_leaf': 5, 'min_samples_split': 2, 'max_features': 3}	0.749(0.644, 0.853)
Decision tree	{'max_depth':range(2,20), 'max_features': range(2,25)， 'criterion':['gini','entropy']， 'splitter': ['best','random'], 'min_samples_leaf':range(1,10), 'min_samples_split':range(2,10)}	{'max_depth': 6, 'max_features': 5, 'criterion': 'gini', 'splitter': 'best', 'min_samples_leaf': 6 'min_samples_split': 2}	0.664(0.488, 0.840)
Logistic Regression	{'penalty': ['l1','l2'], 'dual'[True,False], 'solver': ['liblinear','lbfgs', 'newton-cg','sag','saga'], 'C': [0.01,0.05,0.1,0.5,1,5,10,50], 'fit_intercept': [True,False], 'intercept_scaling':[-0.5,0.5,1,1.5,2]	{‘penalty’='l2', ‘dual’=False, ‘solver’='lbfgs', ‘C’=1, ‘fit_intercept’=True, ‘intercept_scaling’=0.5}	0.717(0.692, 0.743)

	'intercept_scaling':[0,0.5,1,1.5,2]}		
Naive Bayes (GaussianNB)	None	None	0.733(0.650, 0.817)
Support vector machine	{'penalty': ['l2','l1'], 'dual': [True,False], 'loss': ['squared_hinge','hinge'], 'fit_intercept': [True,False], 'C': [0.001,0.005,0.1,0.5,1,5,10]}	{‘penalty ‘= 'l2', ‘dual’=True, ‘fit_intercept’=False, ‘C’=0.1}	0.717(0.687,0.746)
Gradient Boosting	{'n_estimators':range(1,100), 'learning_rate': [0.001,0.005,0.01, 0.05,0.1,0.5,1], 'min_samples_split':range(2,10), 'min_samples_leaf':range(1,10), 'max_features': [range(2,24),’sqrt’], 'max_depth':range(1,100), 'subsample':[0.6,0.7,0.75,0.8, 0.85,0.9,0.95,1]}	{'n_estimators': 31, 'learning_rate': 0.1, 'min_samples_leaf': 7, 'min_samples_split': 2, 'max_features': 'sqrt', 'max_depth': 13, 'subsample': 1}	0.737(0.637,0.838)
MLP	{'neurons':range(6:20),	{'neurons': 15,	0.663(0.532,0.794)

(one layer)	'batch_size':range(4,33), 'nb_epoch':range(10,30), 'lr': [0.001,0.01,0.05, 0.1,0.5,1,2.5,5], 'momentums': [0.0,0.1,0.2,0.3,0.4, 0.5,0.6,0.7,0.8,0.9,1]}	'batch_size': 28, 'nb_epoch': 15, 'lr': 1, 'momentums': 0.8}	

Code available: https://github.com/PigEightZhu/AMI.git

Table S3. Predictive performance of developed machine learning models and logistic regression in the test dataset

Classifier	F1-score	Accuracy	AUC
Logistic regression	0.49	0.55	0.62
Decision tree	0.42	0.57	0.60
Naive Bayes	0.49	0.62	0.64
Support vector machine	0.50	0.57	0.62
Random forest	0.48	0.68	0.68
Gradient boosting	0.42	0.68	0.64
Multilayer perceptron	0	0.69	0.55

Table S4. The Brier score for model calibration

Classifier	Uncalibrated	Calibrated
Logistic regression	0.27	0.24
Decision tree	0.30	0.19
Naive Bayes	0.30	0.23
Support vector machine	0.23	0.23
Random forest	0.21	0.22
Gradient boosting	0.22	0.22
Multilayer perceptron	0.24	0.33

Brier score is defined as the mean squared difference between the observed and the predicted outcome.
